# Reprogramming of Embryonic Human Fibroblasts into Fetal Hematopoietic Progenitors by Fusion with Human Fetal Liver CD34^+^ Cells

**DOI:** 10.1371/journal.pone.0018265

**Published:** 2011-04-14

**Authors:** Vladislav M. Sandler, Nathalie Lailler, Eric E. Bouhassira

**Affiliations:** Division of Hematology, Department of Medicine, Albert Einstein College of Medicine, Bronx, New York, United States of America; University of Frankfurt - University Hospital Frankfurt, Germany

## Abstract

Experiments with somatic cell nuclear transfer, inter-cellular hybrid formation_ENREF_3, and ectopic expression of transcription factors have clearly demonstrated that cell fate can be dramatically altered by changing the epigenetic state of cell nuclei. Here we demonstrate, using chemical fusion, direct reprogramming of the genome of human embryonic fibroblasts (HEF) into the state of human fetal liver hFL CD34+ (hFL) hematopoietic progenitors capable of proliferating and differentiating into multiple hematopoietic lineages. We show that hybrid cells retain their ploidy and can differentiate into several hematopoietic lineages. Hybrid cells follow transcription program of differentiating hFL cells as shown by genome-wide transcription profiling. Using whole-genome single nucleotide polymorphism (SNP) profiling of both donor genomes we demonstrate reprogramming of HEF genome into the state of hFL hematopoietic progenitors. Our results prove that it is possible to convert the fetal somatic cell genome into the state of fetal hematopoietic progenitors by fusion. This suggests a possibility of direct reprogramming of human somatic cells into tissue specific progenitors/stem cells without going all the way back to the embryonic state. Direct reprogramming of terminally differentiated cells into the tissue specific progenitors will likely prove useful for the development of novel cell therapies.

## Introduction

Somatic cells have been reprogrammed into the embryonic state [1], [2], [3], [4], [5], [6], as well as into several types of terminally differentiated cells, including myoblasts [7], macrophages [8], beta-cells [9], and neurons [10]. However, the conversion of the somatic cell genome into a state of tissue-specific stem cells/progenitors has not been demonstrated before now. Embryonic stem (ES) cells can differentiate into many cell types but can remain phenotypically and transcriptionally stable *in vitro* in the appropriate culture conditions. Most terminally differentiated cells are also phenotypically and transcriptionally stable both *in vitro* and *in vivo*. On the contrary, hematopoietic progenitors are uni-, bi-, or multi-potent since they can differentiate into mature blood cells types [11], but have a limited self-renewal capacity and are therefore transcriptionally unstable *in vivo*. While it is possible to prospectively isolate progenitors [12] _ENREF_11 and to demonstrate their differentiation potential they cannot be reliably maintained in undifferentiated state in culture. Here we demonstrate the reprogramming through cell fusion of the genome of human embryonic fibroblasts (HEF) into a transcriptionally unstable state of human fetal liver CD34^+^ (hFL) hematopoietic progenitors capable of proliferating and differentiating into multiple hematopoietic lineages.

## Results

We successfully produced hybrids by fusing HEFs and hFL cells following synchronization of both cell types in metaphase II and knock-down of p53. Knock-down of p53 was tested because of the reported increased efficiency of the reprogramming of somatic cells into induced pluripotency state (iPS) in its absence [13], [14], [15]. Cells were synchronized in mitosis because we hypothesized that the dispersion of transcription factors through the cytoplasm in metaphase as a result of the nuclear membrane break down might contribute to a temporary relaxation of the transcriptional control that defines cell identity and may favor reprogramming [16]. In addition, the synchronization in mitosis excludes the possibility of an incompatibility between the cell cycles of the fusion partners. This is important since such a fusion, for instance, between a cell in mitosis with a cell in interphase would lead to cell death.

**Figure 1:**
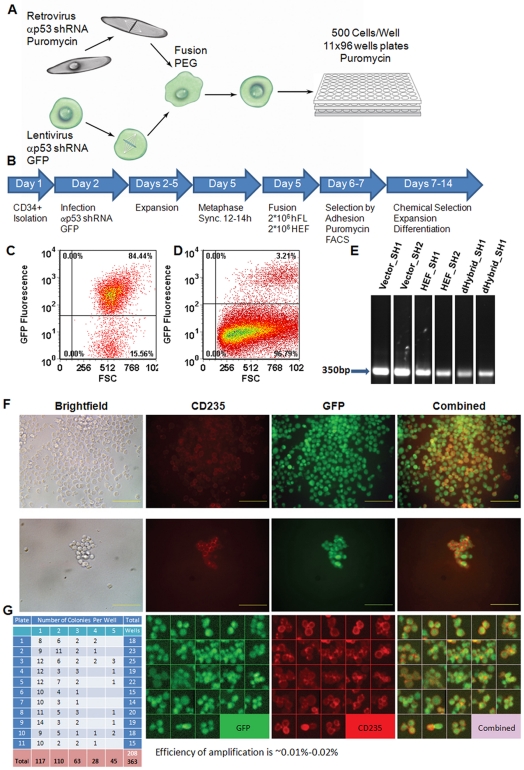
Generation of hybrid cells by fusion of hFL cells and HEFs. **A.** HEFs and hFL cells from two donors were infected with retroviral or lentiviral vectors expressing anti-p53 shRNAs, a drug resistant marker or GFP. Infected cells were synchronized in metaphase, chemically fused, and differentiated towards the erythroid lineage in liquid culture after limiting dilution, and in the presence of antibiotics. **B.** Schematic of the experiment. **C.** FACS analysis demonstrating GFP expression by hFL cells before fusion. **D.** FACS analysis demonstrating GFP expression by hybrid cells 7 days after fusion. **E.** PCR amplification showing that the retroviral vectors used to infect HEFs for p53 knock-down and drug-resistance selection can be detected in the hybrids. **F.** Examples of colonies detected at day 14, the end of the period of expansion, chemical selection, and erythroid differentiation of the hybrid cells. Magnification is ×10 for two upper rows and ×20 for the bottom row. Bottom row shows random computer chosen images of cells from different colonies. **G.** Representative example of the number of hybrid colonies in an experiment.

HEFs were transduced with retroviral vectors expressing anti-p53 shRNAs and a puromycin resistance marker and selected for puromycin resistance. hFL cells were transduced with a lentivirus expressing GFP and anti-p53 shRNA and selected using fluorescence activated cell sorting (FACS) (Fig. 1A, B, C; see Materials and Methods). These two selection markers were then used to select hybrid cells using the scheme described in Fig. 1A and 1B. Briefly, the genetically modified HEF and hFL cells were synchronized in mitosis with the help of nocodazole and chemically fused using polyethylene glycol (PEG). Non-fused HEFs were eliminated, as they were able to attach to the tissue culture plates and did not express GFP. Non-fused hFL cells were selected against chemically using puromycin. Two days after fusion GFP expressing cells (Fig. 1D) were FACS-sorted for GFP and seeded in 96-well plates at the density of 500 cells/well for differentiation toward the erythroid lineage (Fig. 1A, B; see Materials and Methods).

One week after fusion, we observed the emergence of GFP positive, puromycin resistant colonies (Fig. 1F), which ranged in size from 23 cells (Fig. 1F, middle row, the smallest colony observed) to more than 200 cells (Fig. 1F, upper row, the largest colony observed). We detected 363 colonies in 208 wells out of total 1056 wells (Fig. 1G). Assuming that each colony was a progeny of a single hybrid cell, efficiency of successful fusion/amplification was 0.018% (363 cells out of 2*10^6^ hFL cells used for fusion). Observed cells expressed CD235a (Glycophorin A) (Fig. 1F) suggesting that they had differentiated along the erythroid lineage. As expected they were also GFP positive and puromycin resistant.

**Figure 2:**
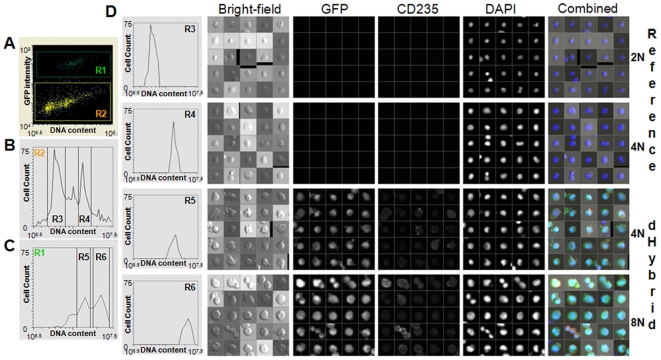
Hybrid cells retain ploidy and differentiate along the erythroid lineage. **A.** GFP expression and DNA content analysis of a mixture of hybrid cells on day 14 of erythroid differentiation and freshly isolated hFL (reference) cells. **B.** DNA content analysis of reference GFP- cells. **C.** DNA content analysis of hybrid GFP+ cells. **D.** Morphology, DNA content, GFP, and CD235 expression analysis of reference diploid and tetraploid cells and hybrid tetraploid and octaploid cells.

To confirm that the puromycin-resistant GFP expressing cells were generated through fusion, we first assayed the genetic markers carried by the donor cells used for the fusion. Using polymerase chain reaction (PCR), we observed that drug-resistant GFP positive cells contained the retroviral insert introduced into the HEFs (Fig. 1E). We then determined the DNA content of the hybrids. We mixed puromycin-resistant GFP-positive cells that went through selection and differentiation in liquid culture (Fig. 1A) with freshly isolated hFL that served as a reference diploid internal control. We then stained the mixture with DAPI and anti-CD235a antibody, and analyzed it using a Laser Scanning Cytometer (LSC) (Fig. 2). The GFP-positive drug-resistant cells contained twice the relative amount of DNA compared to reference cells (Fig. 2B, C), and were tetra-ploid or octa-ploid (Fig. 2C). Single-cell analysis demonstrated that most GFP-positive cells had a higher DNA content compared to the reference cells and were also CD235a positive (Fig. 2D). Together, these experiments confirmed that the fusion was successful.

**Figure 3:**
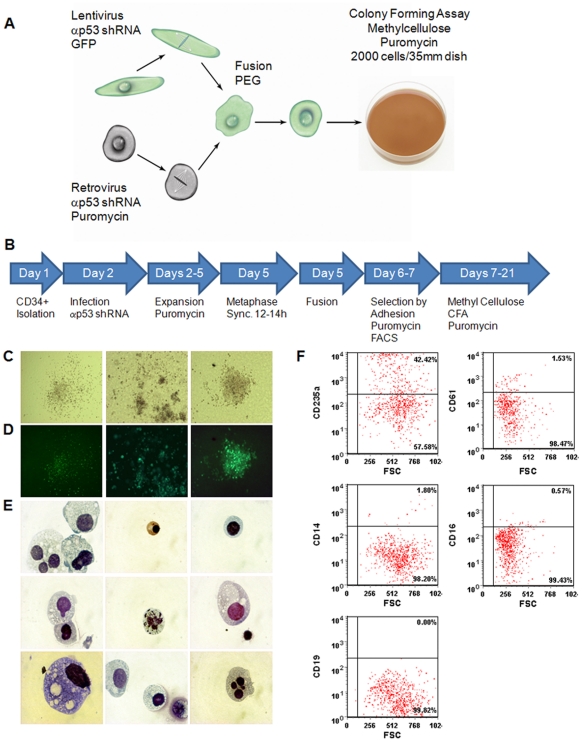
Hybrid cells differentiate into several hematopoietic lineages. **A.** HEFs and hFL cells were respectively infected with lentivirus expressing anti-p53 shRNA and GFP, and retrovirus expressing anti-p53 shRNA and puromycin selection marker. They were synchronized in metaphase, chemically fused, and seeded in methylcellulose medium for colony forming unit assays. **B.** Diagram of the experiment. **C.** Typical colonies that arose in the CFU assay (magnification ×4). **D.** Fluorescent images of the colonies in C. **E.** Wright-Giemsa stain of a cytospin of cells obtained from the CFU assay colonies (magnification ×100). **F.** FACS analysis of cells from the colonies formed in the CFU assay.

If the HEF genome was reprogrammed to the state of a CD34^+^ hFL cell, then the hybrid cells should have retained the multi-lineage potential of hFL cells. To test this assumption we conducted colony forming unit (CFU) assays in methyl-cellulose (Fig. 3); only hFL cells but not HEFs can spontaneously differentiate into different hematopoietic lineages in the assay. Fusion was conducted as described above except that the GFP and puromycin resistance markers were swapped. (Fig. 3A,B; see Materials and Methods). After two days of chemical selection, GFP positive cells were isolated using FACS and seeded in methyl cellulose containing puromycin for CFU assays. The cells gave rise to colonies with morphologies resembling CFU-M, CFU-G, CFU-GM and poorly hemoglobinized BFU-E (Fig. 3C). All colonies were GFP positive suggesting that they contained the genome of the HEFs (Fig. 3D). Identification of the colonies was confirmed by a Wright-Giemsa stain (Fig. 3E). It revealed that that the colonies contained cells with erythroid, macrophage, granulocyte, and megakaryocyte precursors morphologies (Fig. 3E). FACS analysis of these GFP positive colonies revealed a large population of cells expressing CD235a and a much smaller population of monocytes, megakaryocytes, and neutrophils expressing CD14, CD61, and CD16 respectively. As expected, we failed to detect cells expressing CD19, a marker commonly expressed by cells differentiating toward the lymphoid lineage (Fig. 3F).

**Figure 4:**
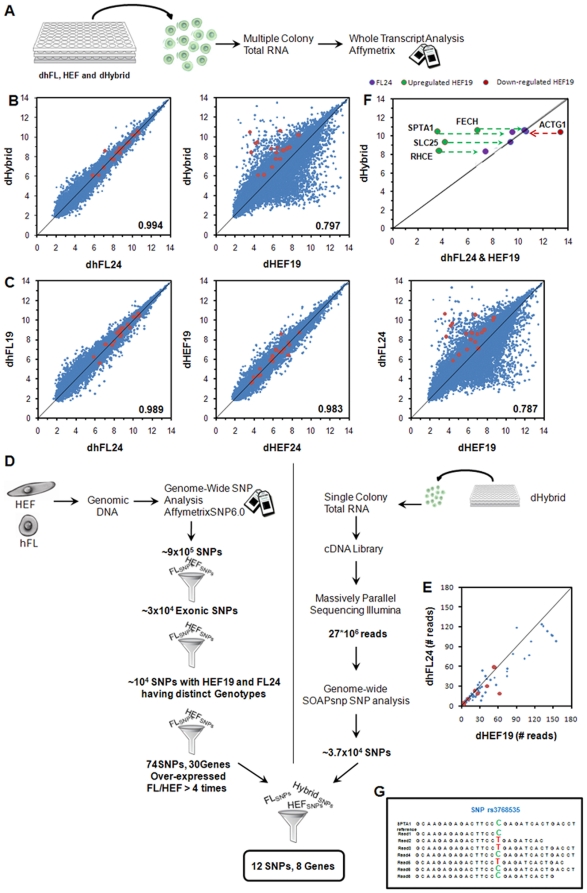
Transcriptional reprogramming of HEF genome in hybrid cells. **A.** Total RNA from about 1500 hybrid cells (12 colonies) and equal numbers of hFL and HEF cells (donors 19 and 24) were analyzed using Affymetrix 1.0 ST Array. **B.** Comparison of genome-wide transcriptional profiles of dHybrids, dhFL cells, and HEFs (donors 19 and 24). **C.** Comparison of genome-wide transcriptional profiles of dhFL and HEF for two donors (donors 19, 24). PMCCs for pair-wise combinations of expression profiles of hybrid cells, hFL cells, and HEFs are shown inside each graph in B and C. The red dots show 18 genes that are highly over-expressed in dhFL and that contain SNPs found using SNP Array and satisfying criteria shown in E (see also Materials and Methods). **D.** An outline of the paradigm that was used to unambiguously identify origin of transcripts in hybrid cells. **E.** Number of reads mapping hybrids SNPs originating from dhFL24 or HEF19 genomes. The red dots show the SNPs from Table 1 having a total number of mapping reads greater than 5. **F.** Change of transcription levels of representative genes expressed by the reprogrammed HEF genome inside hybrid cells. Green dots and arrows show up-regulation of expression of dhFL-specific genes with verified SNPs (see Table 1). A red dot and arrow show down-regulation of HEF specific gene with verified SNP (see Table 1). The scale in all graphs is in log_2_(X). **G.** An example of RNA-Seq analysis for a gene SPTA1 with a partial alignment of Illumina reads with SNPs coming from transcripts of two different genomes (see Table 1, row 6).

We hypothesized that the reprogrammed HEFs should have acquired the transcriptional profile of the hFL fusion partner. To determine whether the hybrid cells resemble the hFL cells, or on the contrary retained the transcription states of both types of fused cells, we performed a genome-wide transcriptional profiling (Fig. 4) using Affymetrix oligonucleotide arrays on HEF cells, hFL cells differentiated along the erythroid lineage (dhFL), and hybrid cells differentiated in the same conditions (dHybrids). Pearson product moment correlations (PMCC) showed that the transcriptional profiles of the hybrid and dhFL cells were closest to each other (PMCC = 0.994) and that the profile of the dHybrids was significantly different from that of the HEF cells (PMCC = 0.797), (Fig. 4B). PMCC between the dHybrid and the dhFL cells (Fig. 4B), and between the dhFL cells from the two donors were similar (Fig. 4B).

The above experiments demonstrated that the hybrid cells had adopted a dhFL transcriptional profile but did not formally prove that the HEF genome was fully reprogrammed since the HEF genome could have been retained as a silent cargo [17], [18], [19]. The extent of reprogramming of the adopted genome in fusion experiments has been a long standing question that can now be answered using massively parallel transcriptome sequencing combined with genome-wide single nucleotide polymorphism (SNP) analysis. To evaluate the extent of the reprogramming of the genome of HEFs in the hybrid cells, we conducted a genome-wide SNP analysis in both donors. We reasoned that (i) if the HEF genome was fully reprogrammed, erythroid-specific genes would be expressed at similar levels whether they were derived from the HEF or hFL genomes and (ii) that fibroblast specific genes would be down-regulated in the hybrids. To test this hypothesis, we first identified SNPs in the genomic DNA of the two donors using an Affymetrix SNP Array (Fig. 4D; see Materials and Methods). We then used a colony of hybrid cells differentiated towards the erythroid lineage (∼100 cells) (Fig. 1F, G) to construct a cDNA library (see Materials and Methods) that was subsequently deep-sequenced on an Illumina GAII genome analyzer. Twenty-seven millions sequence reads were then aligned to the transcriptome and SNPs were called using the SOAP and SOAPsnp packages (see Materials and Methods). To unambiguously identify which genome was the source of a particular transcript, we focused on SNPs that were different in the two donors and either homozygous in both donors or heterozygous in only one donor. We identified 71 statistically robust (mRNA-SEQ read count >5) SNPs in 64 polymorphic genes (Fig. 4E). These genes were found in chromosomes 1, 10, 11, 12, 13, 14, 15, 16, 17, 18, 19, and 22 (Table S1). Quantitative analysis revealed that when the SNPs were homozygous, the ratio of transcripts was 59.6±7.3%/40.4±7.3% (theoretical ideal value 50%/50%). For heterozygous SNPs, the ratio was 73.3±10.3%/26.4±10.3% (theoretical ideal value 75%/25%). To extend this analysis, we then focused on transcripts that were at least 4 times over-expressed in either hFL or HEF cells. We found 11 SNPs in 7 genes over-expressed in hFL and 1 SNP in 1 gene (ACTG1 – actin gamma 1; implicated in hearing loss and cell viability decrease when mutated) over-expressed in HEF that satisfied these criteria (Fig. 4E, F; Table 1). Four out of these 7 genes were known to be hematopoietic (RHCE, SPTA1, LMO2, FECH). Mutations in two of these four genes were implicated in hematopoietic diseases; SPTA1 – spherocytosis, FECH – erythropoietic porphyria. Quantitative analysis revealed that when the SNPs were homozygous, the ratio of HEF-derived/hFL-derived transcripts was 41±3.29%/59±3.29%; theoretical ideal value 50%/50%). For one donor's heterozygous SNPs the ratio of HEF-derived/hFL-derived transcripts was 22±6.3%/78±6.3% (theoretical ideal value 25%/75%; all values are presented as (X ± (Standard Deviation)). These analyses clearly demonstrate that the HEF genome was reprogrammed rather than carried as a silent cargo [18], [19].

**Table 1:** List of SNPs demonstrating effective reprogramming of HEF genome in dHybrid cells.

Chromo some	Position	Gene	Ref. Gen.	Consensus Genome	1^st^ Best Base	1^st^ Best Freq.	2^nd^ Best Base	2^nd^ Best Freq.	HEF19 Genome	hFL24 Genome	Expression dhFL24-HEF19
1	25589895	RHCE	G	G/C	C	53\82	G	28\82	CG	GG	4.62
1	25589952	RHCE	C	G/C	C	40\45	G	5\45	CC	CG	4.62
1	117968400	FAM46C	C	T/C	T	88\112	C	24\112	CT	TT	5.32
1	117968722	FAM46C	A	G/A	G	87\113	A	26\113	GA	GG	5.32
1	156847383	SPTA1	G	G/A	A	45\72	G	27\72	GA	AA	7.1
1	156947511	SPTA1	T	T/C	T	33\45	C	12\45	TT	CT	7.1
11	33837472	LMO2	T	A/T	T	7\10	A	3\10	TT	AT	2.37
11	33837592	LMO2	T	T/C	C	5\8	T	3\8	CC	TC	2.37
14	36219544	SLC25A21	G	G/A	G	4\7	A	3\7	GA	GG	5.24
17	7501560	ATP1B2	T	G/T	T	14\19	G	3\19	N/A	TT	2.84
17	77092614	ACTG1	G	G/A	G	4\7	A	3\7	GA	AA	−3.82
18	53391503	FECH	C	T/C	C	16\21	T	5\21	TC	CC	3.79

SNPS from list A and list B (see Materials and Methods), showing genomic position, the hybrid consensus sequence vs. reference sequence identified using SOAPsnp, frequencies for the 1^st^ and 2^nd^ best base, genome reads for HEFs and hFL cells determined using Affymetrix SNP6.0 array, and differential expression levels for dhFL cells and HEFs according to the RNA-seq analysis. The 1^st^/2^nd^ best base frequency is the count of uniquely aligned reads corroborating the 1^st^/2^nd^ best base divided by the overall sequencing depth of the site. Green and red rows represent genes that are respectively up- and down-regulated in HEF genome inside hybrid cells.

## Discussion

In summary, this work shows that HEF/hFL hybrids proliferate and retain their ploidies for at least 14 days *in vitro*, and are capable of differentiating into several myeloid lineages. The differentiated progeny of the hybrids followed a transcriptional program undistinguishable from the differentiated progeny of the hFL cells suggesting that the hFL genome was unaffected by the HEF genome, and that the HEF genome was completely reprogrammed. It has to be noted that it is yet to be determined if adult human fibroblasts can be reprogrammed into the state of adult multi-potent hematopoietic progenitors.

Our results therefore prove that it is possible to completely convert an embryonic somatic cell genome into a state of fetal liver hematopoietic progenitors without having to first revert to an embryonic state. This suggests that these cells contain factors capable of overriding the factors defining the cellular identity of the HEFs and that it might be possible to identify a set of transcription factors that could be used to directly reprogram somatic cells into tissue specific stem cells/progenitors.

During preparation of this work for publication we learned about the direct conversion of human fibroblasts to multi-lineage blood progenitors by ectopic expression of OCT4 (POU5F1) [20]. This fascinating discovery reconfirms our own conclusions presented here and opens up new opportunities in the area of cellular reprogramming. It remains to be seen whether OCT4 dependent conversion of fibroblasts is tissue type independent and will be useful for generation of adult type erythrocytes as well as common lymphoid progenitors. Direct reprogramming of terminally differentiated cells into tissue-specific progenitors will likely prove useful for the development of novel cell therapies, as well as a better understanding of mechanisms of reprogramming [21], [22], cellular plasticity *in vitro* [22] and *in vivo* [23], as well as of human development.

## Materials and Methods

### Cell Culture

HEFs and hFL CD34+ cells were isolated from discarded tissue of aborted fetuses. All experiments were approved by the Committee on Clinical Investigations (CCI) and conducted according to the CCI approved protocols and written informed consent (English and Spanish) (CCI# 2006-390 “*In vitro* red blood cell production”, and CCI# 2008-201”Feasibility pilot studies of therapies for sickle cell disease, thalassemia and other acquired and inherited blood, vascular and metabolic disorders”). Briefly, fetal liver tissue was mechanically homogenized, trypsinized (0.05% Trypsin solution in PBS) for 20 minutes at 37°C. Mononuclear cells were isolated using gradient centrifugation in HISTOPAQUE-1077 (Sigma-Aldrich, St. Louis, USA). hFL CD34+ cells were isolated using EasySep CD34 Selection Kit (StemCell Technologies, Vancouver, Canada) according to the manufacturer's instructions. HEFs were isolated by trypsinization and mechanical dispersion of fetal tissue. Cell suspension was plated in the cell culture medium made of DMEM, 10% FBS, Pen/Strep. Cells were selected by their ability to adhere to the bottom of the culture dish and proliferate. All experiments were conducted with HEFs younger than passage four.

FL CD34+ cells and hybrid fused cells were expanded, and differentiated in liquid culture as previously described [24]. Briefly, FL CD34+ cells and hybrids were seeded in serum-free basal medium StemSpan (StemCell Technologies, Vancouver, Canada) supplemented with Hydrocortisone (10^−6^ M), SCF 50 ng/ml, Flt3L (16.7 ng/ml), BMP4 (6.7 ng/ml), IL3 (6.7 ng/ml), IL11 (6.7 ng/ml), EPO (1.3 U/ml). After 7 days the concentrations of the cytokines were changed as follows: Hydrocortisone (10^−6^ M), SCF (20 ng/ml), IGF1 (20 ng/ml), IL3 (6.7 ng/ml), IL11 (6.7 ng/ml), EPO (2 U/ml). The medium used for culturing hybrid cells and cells infected with retroviral vectors conferring puromycin resistance was supplemented with puromycin (10 µg/ml).

### Viral vectors

Three different species of short hairpin RNA (shRNA) were used to knock-down expression of p53 in HEFs and FL CD34+ cells, anti-p53 shRNA1, shRNA2, and shRNA3. Anti-p53 shRNA1 was delivered and expressed using lentiviral vector pLVUH-shp53-GFP [25] (Addgene 11653). Anti-p53 shRNA2 and anti-p53 shRNA3 were delivered and expressed using retroviral vectors pMKO.1 puro [26], [27] (Addgene 10671, 10672). Lentiviral and retroviral vectors were packaged as described [25], [28]. Both types of viruses were purified and concentrated using Fast-Trap Lentivirus Purification and Concentration Kit (Millipore, Billerica, USA). Primers used for pMKO.1 puro shRNA2 and shRNA3 detection were as following: pMKO.1_shRNA2_F: 5′-ACT CCT TCT CTA GGC GCC GGA ATT-3′; pMKO.1_shRNA2_B: 5′-CCA CTG TGC TGG CGA ATT CA-3′; pMKO.1_shRNA3_F: 5′-CTT CTC TGG CGC CGG AAT TGA A-3′; pMKO.1_shRNA3_B: 5′-ACC ACT GTG CTG GCG AAT TCA C-3′. Primers were specific for the construct and designed to amplify a fragment of the viral construct of about 350 bp.

### Fusion

Chemical cell fusion was performed using polyethylene glycol (PEG 1500, Roche Applied Science, Indianapolis, USA) according to manufacturer's instructions and as previously described [3], [21]. Briefly, before fusion cells were cultured for 10–12 hours in the presence of 0.1–0.2 µg/ml of nocodazole to arrest and synchronize them in mitosis [29]. For fusion 2*10^6^–1*10^7^ HEFs and FL CD34+ cells were mixed in 15 ml in StemSpan medium, spun down, and drained of supernatant. Broken by gentle agitation cell pellet was slowly mixed with PEG 1500 (50%, 1 ml at 37°C). Total volume of the cell suspension was gradually brought to 10 ml. Cells were spun down, washed twice, and seeded in a puromycin supplemented medium for further experimentation.

### Transcriptional profiling

A genome-wide transcriptional profiling of donor and hybrid cells was performed using human gene Affymetrix 1.0 ST Array according to the manufacturer's protocol. We used the total RNA from about 1500 hybrid cells (12 colonies) and equal numbers of hFL and HEF cells (donors 19 and 24) as starting material for the expression analysis. All signals were normalized using the Robust Multichip Average (RMA) algorithm through RMA Express software. RMA normalization consists of three steps: a background adjustment, a quintile normalization [30], and summarization. Expression profiles for each cell type were correlated against one another using Pearson product-moment correlation coefficients (PMCC). PMCCs were calculated as follows:
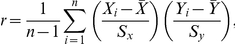
where is standard score, 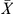 is sample mean, 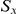 and 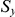 are standard deviations for data sets X and Y respectively.

### Single Nucleotide Polymorphism (SNP) analysis

Genome-wide SNP analysis of donor cells was conducted using Affymetrix SNP Array 6.0. Genomic DNA was extracted from hFL and HEF cells and hybridized on an Affymetrix SNP6.0 array in order to determine SNPs localization and assess the effective reprogramming of the HEF genome in the hybrid cells.

From the total number of identified SNPs, only the ones located within exons were selected, using the Galaxy website (http://main.g2.bx.psu.edu). We used the refseq exons annotation file and looked for the overlap between all exons genomic intervals and SNPs positions. Once the list of exonic SNPs was generated, it was imported into Microsoft Access. Therein, we selected for SNPs that were different in the two donors, and either homozygous in both donors or heterozygous in only one donor. Finally we added a last filtering step allowing us to keep only the SNPs located in genes that were over-expressed at least 4 times in hFL cells when compared to HEFs. We generated a list (List A) of 74 SNPs in 30 Genes.

### Transcriptome-wide SNP analysis

We constructed a cDNA library from total RNA from a single colony of hybrid cells (0.1–1 ng) as described elsewhere [31], [32] _ENREF_27_ENREF_19 with some modifications. We modified primers used for reverse transcription and second strand cDNA synthesis to include a rare-cutter restriction site (BstU1) between unique sequences (UP1 and UP2) and poly (T) fragments of the primers. This allowed us to eliminate UP1 and UP2 anchor sequences from the cDNA library which reduced a number of non-specific reads in RNA-seq. UP1_BstU1 primer: ATA TGG ATC CGG CGC GCC GTC GAC CGC GTT TTT TTT TTT TTT TTT TTT TTT T; UP2_BstU2 primer: ATA TCT CGA GGG CGC GCC GGA TCC CGC GTT TTT TTT TTT TTT TTT TTT TTT T. We increased time of the reverse transcription to 1 hour. Second-strand synthesis was conducted in a two-cycle PCR reaction (95°C for 2 min, 50°C for 3 min, 72°C for 6 min). cDNA amplification was conducted with UP1_BstU1 and UP1_BstU2 primers using 25-cycle PCR reaction (95°C for 30 sec, 68°C for 1 min, 72°C for 6 min plus 6 sec for each consecutive cycle). Amplified cDNA was restricted with the BstU1 endonuclease and purified with Qiaquick PCR purification kit to remove UP1 and UP2 anchor sequences (Figure S1 Materials).

The cDNA library was submitted for Massive Parallel Sequencing using the Illumina GA2x platform. The 27*10^6^ sequences obtained were aligned against the Human genome using SOAP (Short Oligonucleotide Analysis Package) aligner. Finally SOAPsnp, a re-sequencing consensus sequence builder, allowed us to obtain the complete list of 3.7*10^4^ SNPs present in the hybrid exons (List B). Since both donors were females we found SNPs in transcripts of all chromosomes but the Y chromosome (Table S2).

Lists A and B were crossed in order to determine SNPs present in hFL, HEF and hybrid cells. We identified 71 statistically robust (read count >5) SNPs in 64 polymorphous genes regardless of the expression pattern in HEF and hFL. We chose the SNPs that were found in exons of genes that were differentially expressed in hFL when compared to HEF (>4 times difference). We identified 11 SNPs in 7 genes up-regulated in the hybrid cells (hFL specific), and 1 SNPs in 1 genes down-regulated in the hybrid (HEF specific; see Table 1).

## Supporting Information

Figure S1 **Construction of a cDNA library.** **A.** cDNA library constructed from a single colony of hybrid cells. 1.5% agarose gel electrophoresis of the cDNA amplified from a single colony of hybrid cells (0.1–1 ng of RNA). 1/10 of total cDNA library were loaded (lane 1). 10 µl out of 100 µl of total cDNA restricted with BstU1 (lane2). **B.** PCR amplification of fragments of genes over-expressed in dhFL cells from the cDNA library. Lanes 1–8 are show PCR products for NM_004360 (CDH1), NM_005640 (TAF4b), NM_020485 (RHCE), NM_003126 (SPTA1), NM_000347 (SPTB), NM_004091 (E2F2), NM_021624 (HRH4), NM_144682 (SLFN13). Primers for PCR amplification were designed to bind to two different exons of a gene.
(PDF)

Table S1 **List of SNPs and weighted read counts.** SNPs found in the hybrid mRNA sequences, showing genomic position, the hybrid consensus sequence vs. reference sequence identified using SOAPsnp, frequencies for the 1^st^ and 2^nd^ best base, actual genome reads for HEFs and hFL cells determined using Affymetrix SNP6.0 array, and the weighted counts of reads originating from hFL24 or HEF19. When both dhFL24 and HEF19 are homozygous for a gene, the weighting coefficient is 1. If one of the donor is heterozygous for a gene (AB) and the other one is homozygous (AA or BB), a coefficient of 0.66 was attributed to the over-represented allele, and 0.33 to the under-represented allele. The results are shown in the last 2 columns of the table.
(PDF)

Table S2 **Repartition of interrogated genes by chromosome.** The first column contains the number of genes covered by the Affymetrix HuGene.0.1_st micro array for each chromosome, the second columns contains the genes covered in the Affymetrix SNP-6.0 chip, and the last fields shows the number of genes found in the SOAP SNPs analysis run on the hybrid mRNA-seq data.
(PDF)

## References

[1] Gurdon JB, Elsdale TR, Fischberg M (1958). Sexually mature individuals of Xenopus laevis from the transplantation of single somatic nuclei.. Nature.

[2] Eggan K, Baldwin K, Tackett M, Osborne J, Gogos J (2004). Mice cloned from olfactory sensory neurons.. Nature.

[3] Cowan CA, Atienza J, Melton DA, Eggan K (2005). Nuclear reprogramming of somatic cells after fusion with human embryonic stem cells.. Science.

[4] Takahashi K, Yamanaka S (2006). Induction of pluripotent stem cells from mouse embryonic and adult fibroblast cultures by defined factors.. Cell.

[5] Tada M, Takahama Y, Abe K, Nakatsuji N, Tada T (2001). Nuclear reprogramming of somatic cells by in vitro hybridization with ES cells.. Curr Biol.

[6] Ambrosi DJ, Tanasijevic B, Kaur A, Obergfell C, O'Neill RJ (2007). Genome-wide reprogramming in hybrids of somatic cells and embryonic stem cells.. Stem Cells.

[7] Davis RL, Weintraub H, Lassar AB (1987). Expression of a single transfected cDNA converts fibroblasts to myoblasts.. Cell.

[8] Xie H, Ye M, Feng R, Graf T (2004). Stepwise reprogramming of B cells into macrophages.. Cell.

[9] Zhou Q, Brown J, Kanarek A, Rajagopal J, Melton DA (2008). In vivo reprogramming of adult pancreatic exocrine cells to beta-cells.. Nature.

[10] Vierbuchen T, Ostermeier A, Pang ZP, Kokubu Y, Sudhof TC (2010). Direct conversion of fibroblasts to functional neurons by defined factors.. Nature.

[11] Weissman IL, Shizuru JA (2008). The origins of the identification and isolation of hematopoietic stem cells, and their capability to induce donor-specific transplantation tolerance and treat autoimmune diseases.. Blood.

[12] Chao MP, Seita J, Weissman IL (2008). Establishment of a normal hematopoietic and leukemia stem cell hierarchy.. Cold Spring Harb Symp Quant Biol.

[13] Kawamura T, Suzuki J, Wang YV, Menendez S, Morera LB (2009). Linking the p53 tumour suppressor pathway to somatic cell reprogramming.. Nature.

[14] Hong H, Takahashi K, Ichisaka T, Aoi T, Kanagawa O (2009). Suppression of induced pluripotent stem cell generation by the p53-p21 pathway.. Nature.

[15] Li H, Collado M, Villasante A, Strati K, Ortega S (2009). The Ink4/Arf locus is a barrier for iPS cell reprogramming.. Nature.

[16] Egli D, Rosains J, Birkhoff G, Eggan K (2007). Developmental reprogramming after chromosome transfer into mitotic mouse zygotes.. Nature.

[17] Miller RA, Ruddle FH (1976). Pluripotent teratocarcinoma-thymus somatic cell hybrids.. Cell.

[18] Hochedlinger K, Jaenisch R (2006). Nuclear reprogramming and pluripotency.. Nature.

[19] Blau HM, Blakely BT (1999). Plasticity of cell fate: insights from heterokaryons.. Semin Cell Dev Biol.

[20] Szabo E, Rampalli S, Risueno RM, Schnerch A, Mitchell R (2010). Direct conversion of human fibroblasts to multilineage blood progenitors.. Nature.

[21] Bhutani N, Brady JJ, Damian M, Sacco A, Corbel SY (2010). Reprogramming towards pluripotency requires AID-dependent DNA demethylation.. Nature.

[22] Yamanaka S, Blau HM (2010). Nuclear reprogramming to a pluripotent state by three approaches.. Nature.

[23] Johansson CB, Youssef S, Koleckar K, Holbrook C, Doyonnas R (2008). Extensive fusion of haematopoietic cells with Purkinje neurons in response to chronic inflammation.. Nat Cell Biol.

[24] Qiu C, Olivier EN, Velho M, Bouhassira EE (2008). Globin switches in yolk sac-like primitive and fetal-like definitive red blood cells produced from human embryonic stem cells.. Blood.

[25] Szulc J, Wiznerowicz M, Sauvain MO, Trono D, Aebischer P (2006). A versatile tool for conditional gene expression and knockdown.. Nat Methods.

[26] Masutomi K, Yu EY, Khurts S, Ben-Porath I, Currier JL (2003). Telomerase maintains telomere structure in normal human cells.. Cell.

[27] Stewart SA, Dykxhoorn DM, Palliser D, Mizuno H, Yu EY (2003). Lentivirus-delivered stable gene silencing by RNAi in primary cells.. RNA.

[28] Tashiro A, Sandler VM, Toni N, Zhao C, Gage FH (2006). NMDA-receptor-mediated, cell-specific integration of new neurons in adult dentate gyrus.. Nature.

[29] Egli D, Sandler VM, Shinohara ML, Cantor H, Eggan K (2009). Reprogramming after chromosome transfer into mouse blastomeres.. Curr Biol.

[30] Bolstad BM, Irizarry RA, Astrand M, Speed TP (2003). A comparison of normalization methods for high density oligonucleotide array data based on variance and bias.. Bioinformatics.

[31] Kurimoto K, Yabuta Y, Ohinata Y, Saitou M (2007). Global single-cell cDNA amplification to provide a template for representative high-density oligonucleotide microarray analysis.. Nat Protoc.

[32] Tang F, Barbacioru C, Wang Y, Nordman E, Lee C (2009). mRNA-Seq whole-transcriptome analysis of a single cell.. Nat Methods.

