# Supplementary figures and images for: Reprogramming of Embryonic Human Fibroblasts into Fetal Hematopoietic Progenitors by Fusion with Human Fetal Liver CD34+ Cells
Source: PLoS One. 2011 Apr 14;6(4):e18265. doi: 10.1371/journal.pone.0018265 (PMC3077375; doi:10.1371/journal.pone.0018265)

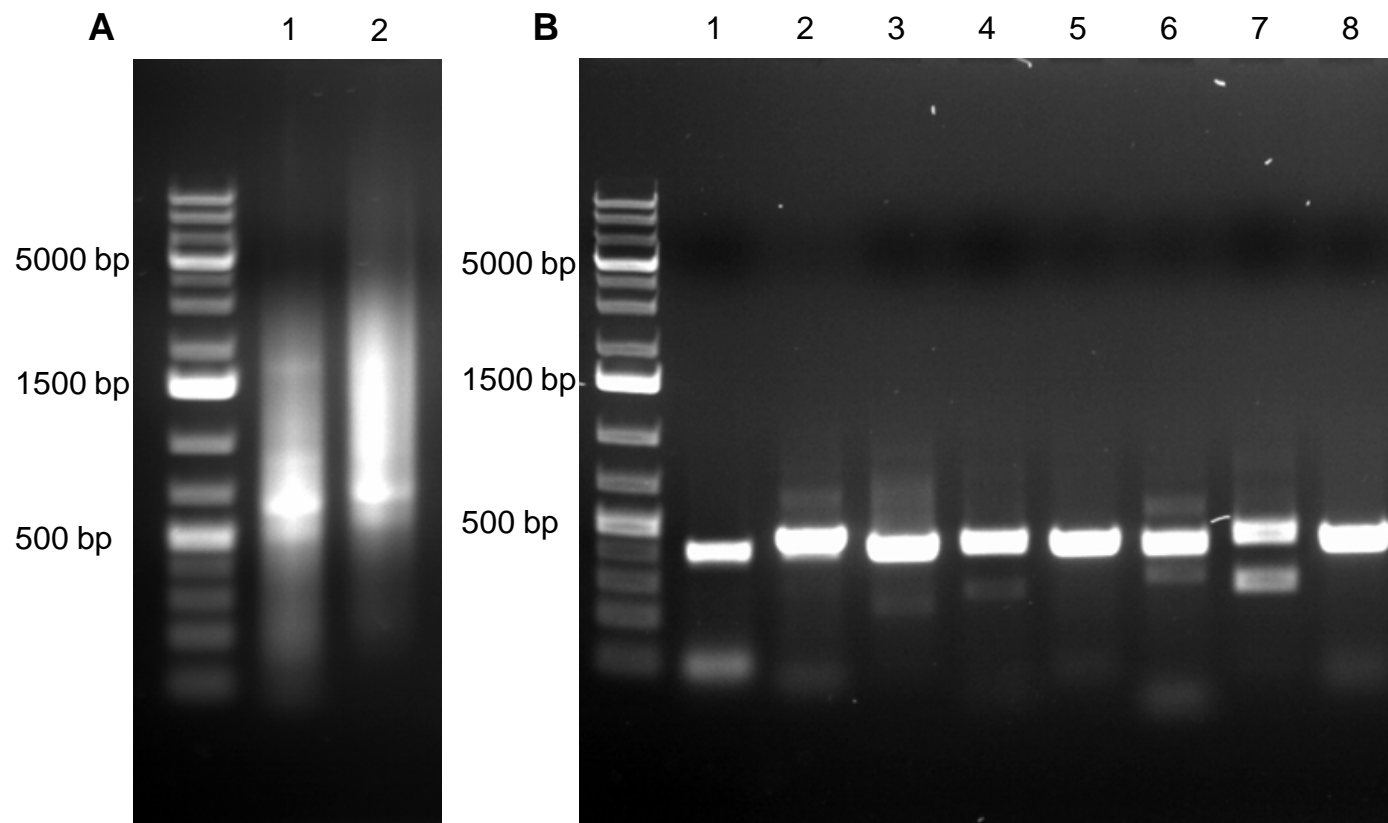

Figure 1 Supplementary

Supplement: Figure S1 — Construction of a cDNA library. A. cDNA library constructed from a single colony of hybrid cells. 1.5% agarose gel electrophoresis of the cDNA amplified from a single colony of hybrid cells (0.1–1 ng of RNA). 1/10 of total cDNA library were loaded (lane 1). 10 µl out of 100 µl of total cDNA restricted with BstU1 (lane2). B. PCR amplification of fragments of genes over-expressed in dhFL cells from the cDNA library. Lanes 1–8 are show PCR products for NM_004360 (CDH1), NM_005640 (TAF4b), NM_020485 (RHCE), NM_003126 (SPTA1), NM_000347 (SPTB), NM_004091 (E2F2), NM_021624 (HRH4), NM_144682 (SLFN13). Primers for PCR amplification were designed to bind to two different exons of a gene. (PDF) [file pone.0018265.s001.pdf]
