# Supplementary material for: Reprogramming of Embryonic Human Fibroblasts into Fetal Hematopoietic Progenitors by Fusion with Human Fetal Liver CD34+ Cells
Source: PLoS One. 2011 Apr 14;6(4):e18265. doi: 10.1371/journal.pone.0018265 (PMC3077375; doi:10.1371/journal.pone.0018265)
Supplement: Table S1 — List of SNPs and weighted read counts. SNPs found in the hybrid mRNA sequences, showing genomic position, the hybrid consensus sequence vs. reference sequence identified using SOAPsnp, frequencies for the 1st and 2nd best base, actual genome reads for HEFs and hFL cells determined using Affymetrix SNP6.0 array, and the weighted counts of reads originating from hFL24 or HEF19. When both dhFL24 and HEF19 are homozygous for a gene, the weighting coefficient is 1. If one of the donor is heterozygous for a gene (AB) and the other one is homozygous (AA or BB), a coefficient of 0.66 was attributed to the over-represented allele, and 0.33 to the under-represented allele. The results are shown in the last 2 columns of the table. (PDF) [file pone.0018265.s002.pdf]

Chromosome	Position	Gene	Reference Genome	Consensus	1stBest base	1stB base frequency	2ndBest base	2nd Best base frequency	HEF19	hFL24	Read Count HEF19	Read count hFL24
chr1	17606151	RCC2	T	G/T	G	21\25	T	4\25	GG	TG	13.86	11.14
chr1	24161063	PNRC2	T	T/C	T	9\15	C	5\15	CT	TT	7.97	6.03
chr1	25422324	SYF2	A	G/A	A	37\51	G	14\51	GA	AA	26.21	24.79
chr1	25589895	RHCE	G	G/C	C	53\82	G	28\82	CG	GG	62.24	18.76
chr1	25589952	RHCE	C	G/C	C	40\45	G	5\45	CC	CG	26.4	18.2
chr1	27145451	NUDC	C	T/C	C	32\42	T	10\42	CT	CC	20.56	21.44
chr1	32281989	KHDRBS1	C	T/C	C	82\97	T	15\97	CC	TC	54.12	42.88
chr1	53288971	SCP2	C	A/C	C	13\27	A	14\27	CA	CC	18.29	8.71
chr1	114912206	BCAS2	A	A/C	C	21\38	A	16\38	AC	CC	22.93	14.07
chr1	117968400	FAM46C	C	T/C	T	88\112	C	24\112	CT	TT	53.04	58.96
chr1	117968722	FAM46C	A	G/A	G	87\113	A	26\113	GA	GG	54.71	58.29
chr1	148885585	GOLPH3L	T	G/T	G	8\14	T	5\14	TG	GG	7.64	5.36
chr1	149049421	ARNT	C	T/C	C	213\695	T	41\695	CC	TC	140.58	113.42
chr1	149638762	PSMB4	G	G/A	A	147\3779	G	106\3779	GA	AA	154.51	98.49
chr1	156847383	SPTA1	G	G/A	A	45\72	G	27\72	GA	AA	41.85	30.15
chr1	156847511	SPTA1	T	T/C	T	33\45	C	12\45	TT	CT	21.78	23.22
chr1	173250256	MRPS14	T	T/C	T	42\48	C	6\48	TT	CT	27.72	20.28
chr1	202658880	PIK3C2B	G	G/A	G	15\21	A	6\21	GG	GA	9.9	11.1
chr1	203322924	RBBP5	A	G/A	A	22\35	G	13\35	GA	AA	20.26	14.74
chr10	16675106	RSU1	A	G/A	A	10\13	G	3\13	GA	AA	6.3	6.7
chr10	106012779	GSTO1	C	A/C	A	115\188	C	73\188	CA	AA	110.95	77.05
chr10	120432872	C10orf46	T	T/C	T	31\50	C	19\50	CT	TT	29.23	20.77
chr11	33688121	CD59	C	G/C	C	23\32	G	9\32	GC	CC	16.59	15.41
chr11	61320875	FEN1	G	G/T	G	25\42	T	17\42	GG	TT	25	17
chr11	74230722	RNF169	C	T/C	T	21\29	C	8\29	TT	CT	13.86	15.14
chr11	74230722	XRRA1	C	T/C	T	21\29	C	8\29	TT	CT	13.86	15.14
chr11	122434085	SPRR1B	C	T/C	C	114\133	T	18\133	CC	CT	75.24	56.76
chr12	15947489	STRAP	A	A/T	A	28\36	T	7\36	TA	AA	16.24	18.76
chr12	16408261	MGST1	G	G/A	G	113\127	A	14\127	GG	AG	74.58	52.42
chr12	24859336	BCAT1	T	T/C	C	80\88	T	8\88	TC	CC	34.4	53.6
chr12	24859398	BCAT1	G	G/A	A	67\84	G	17\84	AG	AA	39.11	44.89
chr12	24859417	BCAT1	A	A/C	C	68\86	A	17\86	AC	CC	39.44	45.56
chr12	24860630	BCAT1	C	G/C	G	177\210	C	32\210	CG	GG	90.41	118.59
chr12	47807939	TUBA1B	G	G/A	G	181\2281	A	74\2281	GA	GG	133.73	121.27
chr12	54406790	CD63	G	G/A	G	146\212	A	66\212	GA	GG	114.18	97.82
chr12	67524002	MDM2	T	T/C	T	21\25	C	4\25	TT	TC	13.86	11.14
chr12	70466439	RAB21	G	G/T	G	59\87	T	28\87	GT	GG	47.47	39.53
chr12	73221010	LOC552889	G	G/T	G	17\21	T	4\21	GG	TG	11.22	9.78
chr12	118608961	CIT	G	G/A	G	53\65	A	12\65	GA	GG	29.49	35.51
chr13	26907031	GTF3A	G	G/C	C	35\51	G	16\51	GC	CC	27.55	23.45
chr13	27137970	POLR1D	G	G/A	A	8\12	G	4\12	AG	AA	6064	5.36
chr13	30803498	B3GALTL	G	G/C	G	13\20	C	7\20	GG	GC	8.58	11.42
chr14	20010355	NP	C	T/C	C	32\40	T	8\40	CC	TT	32	8
chr14	49144270	PPIL5	C	T/C	C	13\16	T	3\16	TC	CC	7.29	8.71
chr14	76995764	AHSA1	C	T/C	C	28\35	T	7\35	CC	TC	18.48	16.52
chr14	103451255	C14orf2	T	T/C	T	107\175	C	68\175	CT	TT	103.31	71.69
chr15	32421325	NOP10	A	G/A	A	159\305	G	96\305	AG	AA	148.47	106.53
chr15	38473142	C15orf23	A	G/A	A	20\32	G	12\32	AG	AA	18.6	13.4
chr16	47275	SNRNP25	T	G/T	G	34\49	T	15\49	GG	TT	34	15
chr16	58741035	GOT2	G	G/A	G	10\12	A	2\12	GG	AG	6.6	5.4
chr16	68844051	AARS	T	A/T	T	10\14	A	4\14	TA	TT	7.3	6.7

Chromosome	Position	Gene	Reference Genome	Consensus	1stBest base	1stB base frequency	2ndBest base	2nd Best base frequency	HEF19	hFL24	Read Count HEF19	Read count hFL24
chr16	82181768	MPHOSP6	A	A/T	A	10\13	T	3\13	AA	TA	6.6	6.4
chr16	84269003	GINS2	T	T/C	T	43\62	C	19\62	CT	TT	33.19	28.81
chr16	84269189	GINS2	G	G/A	G	61\82	A	21\82	AG	GG	41.13	40.87
chr16	84396154	COX4T1	T	T/C	C	161\338	T	94\338	CT	CC	147.13	107.87
chr17	1195142	YWHAE	A	G/A	A	225\12928	G	30\12928	AA	GA	148.5	106.5
chr17	4119692	SNORD115-25	C	A/C	A	48\59	C	11\59	AC	AA	26.84	32.16
chr17	20844368	USP22	T	T/C	T	199\423	C	56\423	TT	TC	131.34	123.66
chr17	31916634	ZNHIT3	A	G/A	G	10\15	A	5\15	GG	AG	6.6	8.4
chr17	37985123	FAM134C	T	A/T	A	45\57	T	12\57	AA	AT	29.7	27.3
chr17	43502806	CBX1	T	A/T	T	18\25	A	7\25	AT	TT	12.94	12.06
chr17	70528216	ICT1	C	T/C	T	31\60	C	29\60	CT	TT	39.23	20.77
chr17	70550341	ATP5H	T	T/C	T	10\14	C	4\14	TC	TT	7.3	6.7
chr17	71449260	ACOX1	T	T/C	C	7\12	T	5\12	CC	TT	7	5
chr17	73682546	TK1	G	G/A	G	48\90	A	41\90	AG	GG	56.84	32.16
chr17	73732603	BIRC5	C	A/C	C	113\121	A	7\121	CC	CA	74.58	45.42
chr18	663016	TYMS	C	T/C	C	39\52	T	13\52	CC	TC	25.74	26.26
chr18	53391503	FECH	C	T/C	C	16\21	T	5\21	TC	CC	10.28	10.72
chr18	53419864	NARS	C	T/C	C	12\20	T	8\20	CT	CC	11.96	8.04
chr19	43564618	PSMD8	C	T/C	C	40\47	T	7\47	CC	TC	26.4	20.6
chr22	37410024	TOMM22	T	T/C	C	84\163	T	79\163	CC	TT	84	79

Table 2 Supplementary
