# Supplementary material for: Reprogramming of Embryonic Human Fibroblasts into Fetal Hematopoietic Progenitors by Fusion with Human Fetal Liver CD34+ Cells
Source: PLoS One. 2011 Apr 14;6(4):e18265. doi: 10.1371/journal.pone.0018265 (PMC3077375; doi:10.1371/journal.pone.0018265)
Supplement: Table S2 — Repartition of interrogated genes by chromosome. The first column contains the number of genes covered by the Affymetrix HuGene.0.1_st micro array for each chromosome, the second columns contains the genes covered in the Affymetrix SNP-6.0 chip, and the last fields shows the number of genes found in the SOAP SNPs analysis run on the hybrid mRNA-seq data. (PDF) [file pone.0018265.s003.pdf]

Table 3 Supplementary

Chromosome	# genes in HuGene.1.0_st array	# of genes in Affymetrix SNP-6.0	#genes in SOAPsnp
1	479	1402	92
2	409	904	45
3	316	844	64
4	251	569	16
5	268	647	32
6	295	737	21
7	266	635	35
8	219	475	17
9	193	535	24
10	201	630	37
11	280	822	31
12	254	711	48
13	141	233	11
14	183	449	24
15	213	445	20
16	170	491	23
17	230	698	74
18	118	264	14
19	138	726	32
20	154	425	35
21	61	184	6
22	88	326	31
X	231	469	25
Y	9	9	0

Table 3
